# Serum profiling by MALDI-TOF mass spectrometry as a diagnostic tool for domoic acid toxicosis in California sea lions

**DOI:** 10.1186/1477-5956-10-18

**Published:** 2012-03-19

**Authors:** Benjamin A Neely, Jennifer L Soper, Denise J Greig, Kevin P Carlin, Elizabeth G Favre, Frances MD Gulland, Jonas S Almeida, Michael G Janech

**Affiliations:** 1 Department of Medicine, Division of Nephrology, Medical University of South Carolina, Charleston, SC, USA; 2 The Marine Mammal Center, Sausalito, CA, USA; 3 National Marine Mammal Foundation, San Diego, CA, USA; 4 Department of Pathology, Division of Informatics, University of Alabama-Birmingham, Birmingham, AL, USA; 5 Research Service, Ralph H. Johnson VA Medical Center, Charleston, SC, USA; 6 Department of Anesthesia and Perioperative Medicine, Medical University of South Carolina, Charleston, SC, USA

**Keywords:** Serum peptides, Neural network, *Zalophus californianus*, Neurotoxin

## Abstract

**Background:**

There are currently no reliable markers of acute domoic acid toxicosis (DAT) for California sea lions. We investigated whether patterns of serum peptides could diagnose acute DAT. Serum peptides were analyzed by MALDI-TOF mass spectrometry from 107 sea lions (acute DAT n = 34; non-DAT n = 73). Artificial neural networks (ANN) were trained using MALDI-TOF data. Individual peaks and neural networks were qualified using an independent test set (n = 20).

**Results:**

No single peak was a good classifier of acute DAT, and ANN models were the best predictors of acute DAT. Performance measures for a single median ANN were: sensitivity, 100%; specificity, 60%; positive predictive value, 71%; negative predictive value, 100%. When 101 ANNs were combined and allowed to vote for the outcome, the performance measures were: sensitivity, 30%; specificity, 100%; positive predictive value, 100%; negative predictive value, 59%.

**Conclusions:**

These results suggest that MALDI-TOF peptide profiling and neural networks can perform either as a highly sensitive (100% negative predictive value) or a highly specific (100% positive predictive value) diagnostic tool for acute DAT. This also suggests that machine learning directed by populations of predictive models offer the ability to modulate the predictive effort into a specific type of error.

## Background

The toxicosis associated with domoic acid (DA) ingestion has been linked to massive marine mammal stranding events along the coastal areas of the Western United States [1]. The California sea lion (*Zalophus californianus*) appears to be the most commonly affected species, likely due to its foraging on fish containing domoic acid [2]. Of sea lions admitted to The Marine Mammal Center (TMMC; Sausalito, CA) between 1998 and 2006 more than 24% were diagnosed with domoic acid toxicosis (DAT), of which 77% were acute DAT [3]. Mortality was significant, regardless of acute or chronic diagnosis, exceeding 40%.

Arriving at a diagnosis of DAT is not always straight forward. Clinical signs can be highly variable [4] and diagnosis often requires post mortem information derived from histological examination of the brain. Some of the variability in clinical signs in stranded animals is likely due to variation in ingested dose, the time at which the sea lion strands, and the time at which examination occurs during the course of the intoxication. Clinical suspicion of DAT is based on the observation of abnormal behavior, *e.g*., ataxia, constant head weaving or seizures [4], which may or may not present at the time of stranding. The diagnosis solely based upon the detection of DA in body fluids or tissues can be misleading because (i) DA is rapidly cleared from the body in experimental animal models [5] and sea lions [6], (ii) the time between ingestion and stranding is unknown, (iii) there is no established harmful dose for sea lions. Blood chemistry and hematology data do not provide information to allow a definitive diagnosis of DAT. High hematocrit, eosinophil counts and serum creatine kinase activities have been reported for sea lions with DAT [4]; however, only creatine kinase activity was found to be abnormal compared with established normal parameters for marine mammals. In a larger study of 715 sea lion cases from 1998 to 2006, abnormal hematological parameters were not found [3] raising the question of utility of these parameters in the differential diagnosis.

Diagnosis of DAT is supported by the finding, after necropsy, of hippocampal atrophy and neuronal necrosis [3,7], although, these changes are not always observed in acutely affected animals. Neuropathologic changes in hippocampal volume measured by magnetic resonance imaging (MRI) or abnormal oscillations in brain electrical activity measured by electroencephalography (EEG) both offer less invasive means to support a diagnosis of chronic DAT [3]. Whether or not MRI or EEG can sufficiently discriminate between living sea lions with acute DAT and stranded sea lions without DAT has not been determined. Regardless, both MRI and EEG are expensive, time consuming, and impractical as a diagnosis tool during mass stranding events [8]. High-throughput, less invasive clinical diagnostic tools for DAT, beyond non-specific changes such as abnormal behavior, which do not require sedation or post-mortem analysis are unavailable. Recently, a semi-quantitative test for habituation to external stimuli was reported to classify sea lions afflicted with DAT with 50% sensitivity and 93% specificity which equates to 81% negative predictive value, and 75% positive predictive value [8]. Although not perfect, a behavioral test such as this does not negatively impact the health of the sea lion and provides support for the diagnosis of DAT.

Time-of-flight mass spectrometry has been used as a clinical discovery tool for a number of human diseases with primary emphasis in cancer [9], but also to classify immunological diseases [10,11], response to erythropoietin therapy [12], renal disease [13], and neurological disorders [14,15] to name a few. The low molecular weight proteome or peptidome found in biological fluids is diverse and changes in the profile can be employed to discriminate between patients with or without disease [16]. In addition, combinations of candidate proteins/peptides can often create a more robust test when individual proteins/peptides fail to discriminate between two groups [17-19]. Large transcriptomic datasets analyzed with machine learning tools were recently shown to enhance the ability to discriminate health status of California sea lions [20]. Because clinical signs and hematological parameters offer little value towards the diagnosis of DAT in sea lions, we investigated whether patterns of serum peptides could offer additional support to the diagnosis of DAT using a similar machine learning approach.

## Results

### Comparison of haematological and clinical parameters

Sera from stranded sea lions collected at TMMC were distributed across multiple years between 2005 and 2010 in proportions equal to 11.5% (n = 10), 6.9% (n = 6), 16.1% (n = 14), 17.2% (n = 15), 35.6% (n = 31) and 12.6% (n = 11), from 2005 to 2010 respectively. Sera from the MMP were collected between 2000 and 2008, and at the time of sampling 7 of 20 exhibited clinical signs which included regurgitation, diarrhea, lameness, vesicles, and abscess from bite wound. Only one animal had prior seizures but clinical signs were unrelated to symptoms associated with DAT. Of the MMP sea lions, 15 of 20 were fasted at the time of collection and 8 of 20 blood samples were taken under anesthesia. Descriptive data available for all individuals in the training dataset is summarized in Additional file 1: Table S1. Individuals in the stranded non-DAT group reflect etiologies common to sea lions admitted to TMMC between 1991 and 2000 [21]; of these 3,379 non-DAT individuals the most prevalent etiologies were malnutrition (35%), leptospirosis (30%), trauma (19%), and miscellaneous (11%) with carcinoma present in 3% of cases [21]. In addition to the 107 animals in the training set, we used an independent test set of 20 animals for qualification, and the identities and diagnoses were blinded to the primary investigator until after analysis. These 20 animals from 2007 to 2010 were chosen by TMMC to include 10 acute-DAT and 10 stranded non-DAT, and in general reflect the types of cases seen in the stranding population. Descriptive data available for individuals in the test set is summarized in Additional file 1: Table S2.

Haematological and serum biochemistry data were available for serum collected from 76 of the 87 individuals in the DAT and stranded non-DAT groups (Table 1) and all 20 individuals in the managed non-DAT group (See Additional file 1: Table S3). Individuals in the acute DAT group had significantly higher red blood cell counts, hemoglobin levels, and hematocrits compared to the stranded non-DAT group or the combined non-DAT group (including MMP sea lions), despite significantly lower levels of BUN and BUN/creatinine ratios (Table 1.). White blood cell counts in the DAT group were 1.7-fold lower compared to the stranded non-DAT group, but this difference was not significant when compared to the combined non-DAT group (*p *> 0.05). Likewise banded neutrophils and lymphocytes were significantly different in the DAT group relative to the stranded non-DAT group (2.9-fold lower and 1.3-fold higher, respectively), but this difference was not significant when compared to the combined non-DAT group (*p *> 0.05). Monocytes were 2.4-fold higher and eosinophils were 3.5-fold higher in sea lions with DAT and both were significantly higher when managed sea lions were included in the analysis. Creatine kinase was significantly higher in the DAT group than the stranded and combined non-DAT groups (1.3 and 1.7-fold higher), and sorbitol dehydrogenase was significantly lower (1.4-fold) than the stranded non-DAT group with no data present for managed non-DAT individuals. Levels of Na, Cl, P, Mg, and Na/K ratios were significantly lower in the DAT group relative to the stranded and combined non-DAT groups (1.1 to 1.0, 1.1, 1.6 to 1.4, 1.3, and 1.1-fold lower, respectively), whereas K was significantly elevated in the DAT group (1.1-fold). Lastly, albumin was significantly higher (1.3-fold) and triglycerides were significantly lower (3.7 -fold) in the DAT group than the stranded non-DAT group but these differences were not significant when managed sea lions were included in the analysis (*p *> 0.05).

**Table 1:** Haematologic and serum biochemistry data of sea lions in the training dataset

Patients with Blood data	n = 29 *Avg ± S.E.*	n = 47 *Avg ± S.E.*	n = 20 *Avg ± S.E.*	DAT v. Stranded non-DAT			DAT v. Combined non-DAT	
**Component**	**DAT**	**Stranded non-DAT**	**Managed non-DAT**	**DAT Fold Change**	***p*-value**	**Combined non-DAT**	**DAT Fold Change**	***p*-value**

**WBC**	11.8 ± 0.5 (n = 29)	19.6 ± 1.6 (n = 42)	6.1 ± 0.6 (n = 20)	-1.7	< 0.001*	15.3 ± 1.3 (n = 62)	-1.3	0.329*

**RBC**	4.9 ± 0.1 (n = 29)	4.3 ± 0.1 (n = 42)	4.4 ± 0.1 (n = 20)	1.1	< 0.001*	4.3 ± 0.1 (n = 62)	1.1	< 0.001*

**HGB**	17.4 ± 0.5 (n = 29)	15.0 ± 0.4 (n = 42)	15.9 ± 0.5 (n = 20)	1.2	< 0.001*	15.3 ± 0.3 (n = 62)	1.1	< 0.001*

**HCT**	51.0 ± 1.4 (n = 29)	43.9 ± 1.1 (n = 42)	45.9 ± 1.5 (n = 20)	1.2	< 0.001*	44.5 ± 0.9 (n = 62)	1.1	< 0.001*

**MCV**	97 ± 4.5 (n = 29)	103 ± 0.8 (n = 42)	104 ± 1.8 (n = 20)	-1.1	0.686*	103 ± 0.8 (n = 62)	-1.1	0.749*

**MCH**	35 ± 0.3 (n = 29)	35 ± 0.3 (n = 42)	36 ± 0.7 (n = 20)	1.0	0.930*	35 ± 0.3 (n = 62)	1.0	0.482

**MCHC**	34 ± 0.2 (n = 29)	34 ± 0.2 (n = 42)	35 ± 0.2 (n = 20)	1.0	0.582*	34 ± 0.1 (n = 62)	1.0	0.502

**PLT**	428 ± 28 (n = 29)	414 ± 24 (n = 42)	358 ± 24 (n = 20)	1.0	0.701	396 ± 18.0 (n = 62)	1.1	0.323

**RDW**	15.8 ± 0.1 (n = 20)	16.2 ± 0.2 (n = 32)	15.3 ± 0.4 (n = 20)	1.0	0.054*	15.9 ± 0.2 (n = 52)	1.0	0.865*

**MPV**	7.7 ± 0.2 (n = 19)	8.1 ± 0.2 (n = 32)	8.7 ± 0.2 (n = 20)	-1.1	0.072	8.4 ± 0.1 (n = 52)	-1.1	0.007

**Segs**	6136 ± 173 (n = 29)	6072 ± 345 (n = 47)	3657 ± 367 (n = 20)	1.0	0.045*	5351 ± 297 (n = 67)	1.1	0.801*

**Bands**	109 ± 44 (n = 29)	311 ± 80 (n = 47)	13 ± 13 (n = 20)	-2.9	0.002*	222 ± 58 (n = 67)	-2.0	0.075*

**Lymphs**	1429 ± 102 (n = 29)	1123 ± 131 (n = 47)	1818 ± 232 (n = 20)	1.3	0.008*	1331 ± 121 (n = 67)	1.1	0.097*

**Monos**	231 ± 39 (n = 29)	97 ± 18 (n = 47)	389 ± 86 (n = 20)	2.4	< 0.001*	184 ± 33 (n = 67)	1.3	0.079*

**Eosin**	868 ± 115 (n = 29)	247 ± 59 (n = 47)	214 ± 62 (n = 20)	3.5	< 0.001*	237 ± 45 (n = 67)	3.7	< 0.001*

**Na**	148 ± 0.9 (n = 28)	156 ± 1.6 (n = 46)	149 ± 0.5 (n = 20)	-1.1	< 0.001*	154 ± 1.2 (n = 66)	-1.0	< 0.001*

**K**	4.7 ± 0.09 (n = 28)	4.3 ± 0.10 (n = 47)	4.3 ± 0.10 (n = 20)	1.1	0.026	4.3 ± 0.08 (n = 67)	1.1	0.012

**Cl**	109 ± 0.7 (n = 28)	119 ± 1.6 (n = 47)	108 ± 0.8 (n = 20)	-1.1	< 0.001*	115 ± 1.3 (n = 67)	-1.1	0.002*

**BUN**	21 ± 2.9 (n = 28)	100 ± 15.7 (n = 47)	26 ± 1.3 (n = 20)	-4.8	< 0.001*	78 ± 11.7 (n = 67)	-3.7	< 0.001*

**Creatinine**	0.7 ± 0.04 (n = 28)	3.1 ± 0.73 (n = 47)	1.1 ± 0.08 (n = 20)	-4.4	0.260*	2.5 ± 0.52 (n = 67)	-3.6	0.016*

**Glucose**	108 ± 4.9 (n = 28)	104 ± 4.0 (n = 47)	125 ± 5.2 (n = 20)	1.0	0.948*	110 ± 3.4 (n = 67)	1.0	0.394*

**Ca**	8.3 ± 0.12 (n = 27)	8.0 ± 0.13 (n = 47)	9.6 ± 0.12 (n = 20)	1.0	0.091*	8.5 ± 0.14 (n = 67)	1.0	0.405

**P**	5.1 ± 0.3 (n = 28)	8.0 ± 0.5 (n = 47)	5.7 ± 0.3 (n = 20)	-1.6	< 0.001*	7.3 ± 0.4 (n = 67)	-1.4	< 0.001*

**Total Protein**	7.8 ± 0.15 (n = 28)	7.8 ± 0.18 (n = 47)	7.3 ± 0.23 (n = 20)	1.0	0.771*	7.6 ± 0.14 (n = 67)	1.0	0.213*

**Albumin**	3.0 ± 0.11 (n = 28)	2.4 ± 0.08 (n = 47)	3.4 ± 0.05 (n = 19)	1.3	< 0.001	2.7 ± 0.08 (n = 66)	1.1	0.370

**Bilirubin Total**	0.5 ± 0.07 (n = 28)	0.7 ± 0.18 (n = 47)	0.2 ± 0.03 (n = 20)	-1.4	0.774*	0.5 ± 0.13 (n = 67)	1.0	0.038*

**GGT**	116 ± 11 (n = 27)	217 ± 25 (n = 47)	67 ± 7 (n = 20)	-1.9	< 0.001*	172 ± 20 (n = 67)	-1.5	0.232*

**AST (SGOT)**	65 ± 12 (n = 28)	62 ± 11 (n = 46)	21 ± 2 (n = 20)	1.0	0.438*	49 ± 8 (n = 66)	1.3	0.045*

**ALT (SGPT)**	88 ± 13 (n = 28)	74 ± 8 (n = 47)	38 ± 3 (n = 20)	1.2	0.095*	63 ± 6 (n = 67)	1.4	0.002*

**Alkaline Phosphatase**	59 ± 23 (n = 27)	50 ± 10 (n = 47)	98 ± 9 (n = 20)	1.2	0.796*	64 ± 8 (n = 67)	-1.1	0.081*

**Fe**	86 ± 7 (n = 24)	94 ± 6 (n = 46)	115 ± 8 (n = 20)	-1.1	0.470	100 ± 5 (n = 66)	-1.2	0.150

**Cholesterol**	172 ± 9 (n = 28)	178 ± 10 (n = 47)	334 ± 18 (n = 20)	1.0	0.827*	224 ± 13 (n = 67)	-1.3	0.058*

**Triglycerides**	52 ± 4 (n = 28)	191 ± 35 (n = 47)	31 ± 4 (n = 20)	-3.7	0.003*	143 ± 26 (n = 67)	-2.8	0.493*

**Mg**	1.9 ± 0.07 (n = 23)	2.5 ± 0.17 (n = 44)	2.6 (n = 1)	-1.3	0.019*	2.5 ± 0.17 (n = 45)	-1.3	0.014*

**Creatine Kinase**	1881 ± 417 (n = 27)	1434 ± 571 (n = 46)	341 ± 101 (n = 20)	1.3	0.070*	1103 ± 403 (n = 66)	1.7	0.003*

**BUN/Cr Ratio**	30 ± 4 (n = 28)	55 ± 6 (n = 47)	29 ± 5 (n = 20)	-1.8	< 0.001*	47 ± 5 (n = 67)	-1.6	0.002*

**Na/K Ratio**	32 ± 0.7 (n = 28)	36 ± 1.4 (n = 47)	35 ± 0.7 (n = 20)	-1.1	0.002*	36 ± 1.0 (n = 67)	-1.1	< 0.001*

**SDH (Sorbitol Dehydrogenase)**	21 ± 4 (n = 20)	30 ± 4 (n = 39)	-	-1.4	0.016*	n.a.	n.a.	n.a.

A *T*-test (or rank sum test if distribution was non-normal, indicated by '*') was used to compare blood chemistry values between groups

### MALDI-TOF profiling

**Figure 1:**
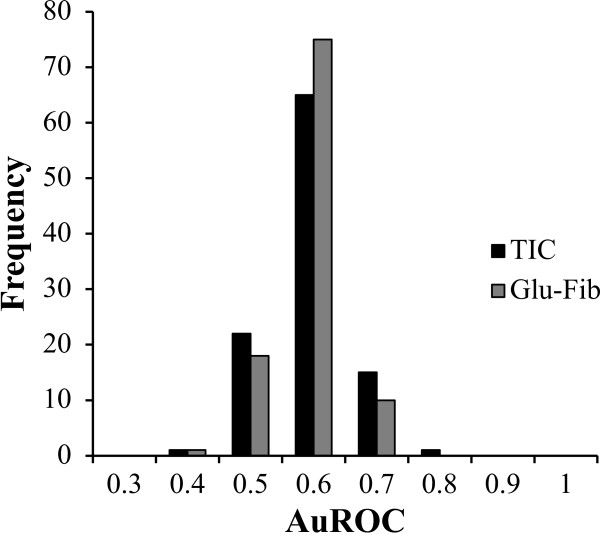
**Distribution of AuROC for 104 peaks normalized to Glu-Fib or TIC**. Receiver operator characteristic curves were constructed using peaks normalized to either Glu-Fib or TIC. Area under the ROC curve (AuROC) was calculated for 104 peaks and binned every 0.1 ± 0.05.

**Figure 2:**
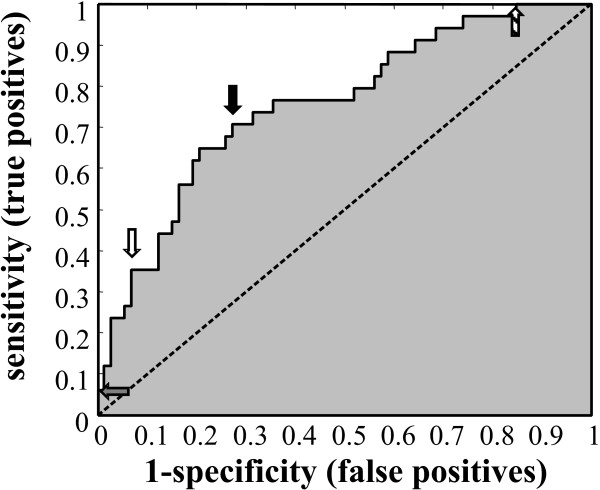
**Receiver operator characteristic curve for peak 3017 m/z**. The TIC normalized training dataset was used to generate an ROC curve with an AuROC ± S.E. of 0.754 ± 0.054. Four thresholds are shown by arrows: minimum mis-classified cut-off (hollow arrow), optimum threshold (solid arrow), negative predictive value cut-off (striped arrow), and positive predictive value cut-off (grey arrow).

Processing of MALDI-TOF generated spectra resulted in the selection of 104 individual peaks that were used for biomarker analysis. To determine if a single peak could discriminate between the DAT and non-DAT group, receiver operator characteristic (ROC) curves were generated using normalized peak height. No single peak had area under the curve (AuROC) > 0.8, therefore none were deemed excellent classifiers of DAT (Figure 1). Peaks normalized using Glu-Fib had a mean AuROC of 0.543, ranging from 0.383 to 0.692. TIC normalized peaks had a mean AuROC of 0.538, ranging from 0.396 to 0.754. The best individual classifier was peak 3017 m/z (TIC normalized) which had an AuROC ± S.E. of 0.754 ± 0.054 (Figure 2). When the independent test set was used to qualify the performance of peak 3017 m/z with a threshold determined by minimal-misclassification the test achieved 100% specificity but only 20% sensitivity with 8 of 10 acute-DAT individuals being called incorrectly (Table 2; Additional file 2: Table S4).

**Table 2:** Qualified performance of models using an independent test set

		3017 m/z	**ANN**_**1**_	**ANN**_**67**_	**ANN**_**67**_	**ANN**_**53**_	CANN-Vote
	**Normalization**	TIC	TIC	TIC	TIC	Glu-Fib	Glu-Fib

	**AuROC**	0.7538	0.9412	0.9412	0.9412	0.9428	na

	**Threshold^*a*^**	minMC	OpT	OpT	minMC	OpT	> 50 votes

**Test Set**	**sensitivity**	0.20	1.00	0.40	0.70	0.30	0.30
	
	**specificity**	1.00	0.60	0.90	0.90	1.00	1.00
	
	**PPV**	1.00	0.71	0.80	0.88	1.00	1.00
	
	**NPV**	0.56	1.00	0.60	0.75	0.59	0.59

**Training Set**	**sensitivity**	0.35	0.91	0.88	0.91	0.94	1.00
	
	**specificity**	0.93	0.95	1.00	0.99	0.96	0.99
	
	**PPV**	0.71	0.89	1.00	0.97	0.91	0.97
	
	**NPV**	0.76	0.96	0.95	0.96	0.97	1.00

^***a ***^Thresholds calculated *a priori *based on training set data. minMC, minimum mis-classified threshold; OpT, optimum threshold (OpT).

Performance against training set is given for comparison

In addition to evaluating individual peak performance for predicting DAT, we were also interested in whether there were peaks that predicted individuals in the managed non-DAT group comprised of sea lions under long-term care of the MMP. When performance was evaluated for discriminating managed non-DAT versus stranded individuals, 21 TIC normalized peaks had an AuROC > 0.8 and the best performer, 1362 m/z, had an AuROC ± S.E. of 0.979 ± 0.023 (see Additional file 3: Figure S1). Interestingly this peak was mostly absent in stranded sea lion sera. Using the OpT threshold, the individuals in the managed non-DAT group were called correctly 18 of 20 times (90% sensitivity), and of the two mis-called sea lions (#2 and #9), sea lion #2 showed clinical signs (behavioral; poor performance). Moreover only four stranded individuals (CSL 6896, 9111, 9271 and 9770) were called incorrectly (95% specificity). Using this same threshold only one individual in the independent test set was called managed non-DAT (CSL 9766, an adult female with acute DAT which had recovered and was ultimately released).

**Figure 3:**
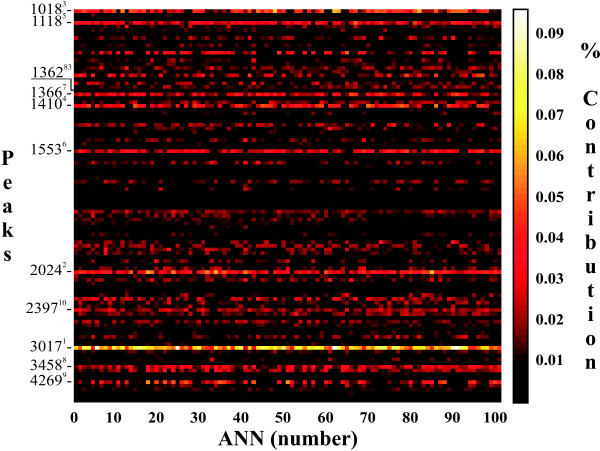
**Relative contribution of each MALDI-TOF peak to each of the 101 ANNs trained using TIC normalized data**. The peaks with the 10 highest average percent contribution are indicated, with superscripted rank. Peak 1362 m/z is indicated for reference.

Because no single peak was an excellent classifier of DAT, unsupervised hierarchal clustering was used to determine whether multiple peaks could separate DAT from non-DAT sea lions (See Additional file 4: Figure S2). The two groups did not form separate clusters, suggesting unsupervised methods are not capable of discriminating the complex relationship between DAT and non-DAT. Therefore peak data were modeled using a supervised informatic method: feed-forward artificial neural networks (ANNs). Glu-Fib and TIC normalized peaks were used separately, and 101 ANNs were trained. In addition to using individual ANNs, we also combined all 101 ANNs and allowed each to vote for the outcome (CANN-vote), or simply averaged all 101 predictions (CANN_101_). The generated models were qualified using a blinded independent test set of 20 sera from stranded sea lions, which were diagnosed with acute DAT (n = 10) or non-DAT (n = 10). Using decision thresholds determined *a priori*, the performance of each model was evaluated for each of the 20 sea lions (Table 2). Compared to the single peak 3017 m/z which gave 100% specificity but only 20% sensitivity, testing different individual ANNs we achieved high specificity (100%) or high sensitivity (100%). Specifically, we found the best performance of Glu-Fib normalized data was 30% sensitivity and 100% specificity, which was achieved using a median ANN (Glu-Fib-ANN_53_). Relative to ANNs trained on Glu-Fib normalized data, models trained using TIC normalized data achieved higher sensitivity (100% versus 40%) as well as high specificity (90%; Supporting information Tables S5 and S6). A negative predictive value of 100% was achieved using a median ANN (TIC-ANN_1_) which was the highest seen in any model (Table 2). This model predicted all 10 DAT individuals correctly with four false positives. The four individuals that were predicted incorrectly could not be explained by sex, age, primary etiology or blood chemistry (See Additional file 2: Table S4). It was also interesting to note that peak 3017 m/z was a large contributor to ANNs trained on TIC normalized data (Figure 3). We also observed that other median TIC ANNs (*i.e*., ANNs with AuROCs equal to the median) had different performance measures despite the same AuROC (*e.g*., TIC-ANN_1 _versus TIC-ANN_67_; Table 2), and overall using the OpT threshold when different from the minMC threshold resulted in higher sensitivity with minimum loss of specificity. For example, in the case of TIC-ANN_67_, the OpT improved performance while maintaining the same specificity as the minMC (Table 2). When allowed to vote, we achieved 100% specificity and 30% sensitivity (Glu-Fib-CANN-vote; Table 2), while other CANN models did not perform as well when 100% specificity was achieved (Supporting Information Tables S5 and S6).

## Discussion

In this study we show patterns of serum peptides can be used to discriminate between stranded sea lions afflicted with acute DAT and those unaffected, with excellent positive or negative predictive value (*i.e*., 100%). Currently DAT is diagnosed based on a variety of clinical signs [3,4,22] as well as the presence of domoic acid in body fluids [1,4], but is generally only confirmed post-mortem [7,8,22]. Moreover, individuals are further characterized along a continuum of toxicosis ranging from acute to chronic, with the former recovering over time and the latter progressing to *status epilepticus *[3,4,22]. The symptomology separating acute from chronic DAT can be inconclusive, such as inconsistent MRI-observable hippocampal atrophy associated with chronic DAT [3,7,22,23]. As a diagnostic tool MRI is expensive, requires sedation and is impractical during a mass stranding event [8]. Overall, the lack of a clear gold-standard for diagnosing DAT highlights the complexity of the underlying physiopathology. Additionally, individuals on the far end of the spectrum, chronic DAT, are poor rehabilitation candidates and often euthanized [22]. Therefore the best test would be one that is rapid, accurate (minimizing false positives), and high-throughput to allow for immediate testing even in the absence of abnormal behavior.

Peptide profiling by MALDI-TOF is a high-throughput tool which is sensitive across a large dynamic range [24] and has been used to classify diseases in humans [25]. A major concern with any biomarker study is experimental design [18], therefore we focused on three areas of likely variability: DAT diagnosis, sample handling, and case-controls. For our training set we used sera from individuals that were diagnosed with acute DAT (according to [3,22]) without confounding etiologies and rejected non-DAT individuals with signs of DAT (acute or chronic). We also chose to not use pregnant females since we assumed their blood peptide levels would be greatly influenced by pregnancy. Pregnant females are a key group to be addressed in future research since studies suggest pregnant females are spatially more likely to be exposed to domoic acid [26] and approximately 25% of females admitted between 1998 and 2006 had evidence of reproductive failure [3]. To account for technical variability due to serum handling, specifically the effect of serum clotting time on peptide profiles [19,27], only sera banked at -80°C on the day of collection were utilized. In addition, sea lions were frequency matched to control for differences in age and sex. Haematologic comparisons of stranded sea lions with acute DAT and those without showed that individuals with acute DAT have significantly increased hematocrit, eosinophil counts, and levels of creatine kinase in congruence with previous reports [4,28]. Higher hematocrit may indicate dehydration although individuals with acute DAT had significantly lower BUN and BUN/creatinine ratios that aligned with managed non-DAT individuals suggesting other influences aside from dehydration could affect hematocrit. The clinical use of BUN and BUN/creatinine ratio in the context of domoic acid toxicosis has not been well described, but monosodium glutamate administration in rats has been shown to decrease serum BUN and creatinine while elevating BUN/creatinine ratio [29]. We also found that acute DAT individuals had a 1.7 to 1.3-fold reduction in white blood cell counts compared to non-DAT individuals, though these values are still within reported normal ranges (9.4 to 22.8; [30]). This is contrary to reports of dolphins exposed to DA in which there was a concurrent eosinophil and white blood cell increase [31]. Although haematology and serum biochemistry are useful for narrowing the diagnosis, alone they cannot confirm specific etiologies. We postulate that these broad differences may indicate an underlying pathology specific to domoic acid exposure (*e.g*., neuronal necrosis and eosinophilia).

Using MALDI-TOF we selected 104 peaks in the training set of 107 individuals to be used for calculating different thresholds *a priori *for single markers and models based on different performance goals, *e.g*., high specificity while minimizing false positives. Performance was qualified using an independent test set since it is known that cross-validation alone overestimates model performance [18]. Since these peaks reflect a fraction of the peptidome and any changes reflect changes in the body due to the nature of blood circulation, we hypothesized that there would be a peptide or group of peptides that could discriminate sea lions with DAT, but no single peak was a good discriminator of DAT (AuROC > 0.8). Interestingly, individuals in the managed non-DAT group could be largely classified based on the absence of peak 1362 m/z with only two managed individuals being misclassified. Furthermore, peak 1362 m/z was able to largely discriminate the stranded population from the managed population, with only five stranded individuals being classified incorrectly. Because the managed population is comprised solely of male sea lions it is possible that 1362 m/z is a sex specific marker; however four out of five misclassified sea lions from the stranding population were female thereby refuting this idea. While we contend that this peak may be a marker of stress it is also possible that differences in sample handling may also influence the presence of this peak [19,27]. Given the differences in peak 1362 m/z between the managed and stranded populations we feel additional studies are warranted to determine the identity and utility of this peptide.

Because no single peak was an excellent classifier of DAT or non-DAT, neural network models were constructed to interrogate multidimensional relationships. Previous reports have demonstrated that more than one classifier can increase test performance, because a panel of biomarkers is more robust to inherent biological perturbation and individual variation is mitigated by the group [19,20,32]. Similarly, we found that artificial neural networks trained using all 104 peaks outperformed single classifiers based on increased sensitivity. The model that gave the highest sensitivity (100%) and specificity (60%), TIC-ANN_1_, only mis-classified four individuals in the test set as DAT. This could not be explained by sex, age, primary etiology or blood chemistry, indicating the model is not overtly biased. Interestingly the most weighted peak in resulting ANNs was also the best single classifier of DAT (3016 m/z) highlighting the potential importance of this peak as a classifier. Lastly, our results highlight the importance of evaluating different decision thresholds, which can improve performance and sensitivity while maintaining specificity. This may prove invaluable in diagnosing DAT since minimizing false positives and maintaining acceptable sensitivity will facilitate diagnosis and treatment.

## Conclusions

Despite the need for a highly specific test for DAT, currently there is little information on the accuracy of current DAT diagnostic approaches. We achieved 100% sensitivity and 60% specificity in a single model (TIC-ANN_1_) and demonstrated that MALDI-TOF peptide profiling and neural networks together can perform as a highly sensitive (with 100% NPV) or highly specific (with 100% PPV) diagnostic tool for acute DAT. Although the test set NPV is 100% for TIC-ANN_1_, the training set NPV at the same threshold suggests that the true NPV for this test is likely 96% (Table 2). This technique also has the potential to be used as a high-throughput diagnostic tool to allow for immediate testing even in the absence of abnormal behavior, although due to the technical nature of the process it is less likely to be a point-of-care test and better suited for a centralized laboratory. Future studies will also address the usefulness of combined peptidomic or proteomic analysis to discriminate sea lions with chronic DAT from sea lions with acute DAT, which has increased in prevalence in recent years [3]. Since samples can be drawn at the time of admission, a prognostic test may be developed to place individuals in risk categories and/or diagnosis on the continuum of acute to chronic DAT based on negative outcome of individuals over time. Techniques such as these may also provide insight into the biology of progression from acute to chronic DAT, or markers of DA exposure in individuals without identifiable clinical signs. In conclusion, this test highlights the benefits and potential of using MALDI-TOF peptide profiling as an accurate, rapid, non-invasive, robust tool to identify sea lions with acute DAT.

## Methods

### Serum collection and storage

Samples were collected by The Marine Mammal Center (TMMC; Sausalito, CA) under IACUC approved protocols and under the National Fisheries Service permit number 932-1489-00 and by the U.S. Navy Marine Mammal Program (MMP; San Diego, CA) as part of a standard clinical care regime. At both locations, blood samples were collected in serum separator tubes (SST) and allowed to clot for 30 to 60 min, centrifuged, serum transferred into cryovials, and stored at -80°C. Less than 7 h passed between clotting and storage at -80°C. Since it has been shown that the number of freeze thaw cycles can affect the measured peptide profile of frozen sera [33], all samples were thawed at the Medical University of South Carolina at 37°C for one min, then placed on ice and 60 to 110 μL aliquots were stored at -80°C. Hematology and serum biochemistry data were included for comparison if the data corresponded to the same day as the drawn sample.

### Inclusion criteria

Individuals in this study were divided into three groups: those suffering from acute domoic acid toxicosis (acute DAT), individuals admitted to TMMC that were asymptomatic for DAT (stranded non-DAT), and individuals from the MMP that were asymptomatic for DAT (managed non-DAT). Except for one sea lion that had just been admitted into the MMP from TMMC, all MMP individuals had been under the long-term care of veterinarians, hence were collectively deemed 'managed'. The remaining two groups, acute DAT and stranded non-DAT were sampled at TMMC and were defined using available clinical parameters. We retrospectively identified serum samples from California sea lions that stranded alive along the central California coast between 2005 and 2010 (n = 2343). Of these, sera drawn within seven days of admission to TMMC were allowed (~2000). We included sera from both sexes and adult, subadult, juvenile, and yearling age classes (as determined by [21]). Individuals were included in the acute DAT group based on clinical signs such as seizures or neurological clinical signs [4] and in some cases the presence of domoic acid in bodily fluids. Specifically, acute DAT cases were differentiated from individuals with chronic DAT as described by Goldstein et al. [3] or with available brain histology (hippocampal atrophy indicated chronic DAT; [7]). Individuals included in the stranded non-DAT group stranded for reasons other than DAT.

### Exclusion criteria

Sera from known pregnant females (*i.e*., those that aborted later in rehabilitation or with a fetus in uterus at necropsy), individuals with significant trauma (*e.g*., missing limbs or life threatening wounds), or individuals later diagnosed with chronic DAT were excluded from the study. Sera were excluded if collected more than seven days after admission, by heart-stick, post-mortem, or if they were not archived at -80°C the day of collection. Individuals were excluded from the acute DAT group if there was a confounding etiology (*e.g*., carcinoma or leptospirosis). Leptospirosis was diagnosed based on blood chemistry or at necropsy (as described in [21]). Five individuals in the acute DAT group were missing hematology data but had no indications of leptospirosis (*e.g*., post-mortem kidney changes characteristic of leptospirosis [21]). Conversely, individuals were excluded from the stranded non-DAT group if seizures/other neurological problems were observed during their time in rehabilitation (regardless of etiology), histology indicated brain morphological changes consistent with DAT, they were positive for DA in bodily fluids, or if they later stranded with signs of DAT.

### Experimental design

A training sample set was analyzed followed by an independent test set used to qualify biomarker and model performance. The training set (n = 107) was comprised of nearly all acute DAT sera samples available from TMMC meeting the criteria described above, frequency matched non-DAT sea lions, and non-frequency matched managed samples from the MMP (See Additional file 1: Table S1). The independent test set (n = 20) was blinded to Medical University of South Carolina personnel processing the samples and analyzing the data. Sea lion samples included in the test set were randomly chosen from the TMMC acute DAT and non-DAT sample population prior to analysis of the training set. Test set samples were processed and analyzed on a day that was different than training set samples. Furthermore, the test set was shipped separately from the training set to avoid any handling bias between training and test set samples. Sera from the training set were extracted and analyzed on two different days. To limit the effect of interday MALDI-TOF variability [27], the training set was divided into two groups (Day 1 or Day 2) that had equal sample proportions representing acute DAT, stranded non-DAT, and managed sea lions. Each group was equally represented for sex, age class, draw year, and outcome (release or euthanasia/death).

### MALDI-TOF

Immediately before being processed, serum aliquots were thawed at 37°C for one min after which 50 μL was transferred to a 200 μL PCR tube. Additional freeze-thaws were not allowed. Next, each serum was diluted to 0.1% (v/v) TFA using 100 μL of 0.15% (v/v) TFA (Thermo Scientific, Rockford, IL) and incubated at room temperature for 5 min prior to the addition of 10 μL C8-magnetic beads (ClinProt™ Profiling Kit, Bruker Daltonics, Billerica, MA). Magnetic beads and serum were incubated for 1 min at room temperature, followed by three wash steps of 100 μL 0.1% (v/v) TFA according to manufacturer's guidelines. Peptides were eluted with 20 μL of 50% acetonitrile in stabilization buffer (Bruker Daltonics) and 15 μL was transferred to a clean tube. Finally, 30 μL of matrix [5 mg mL^-1 ^α-cyano-4-hydroxycinnamic acid (Bruker Daltonics) in HPLC grade methanol:acetonitrile:water (5:4:1) containing 25 nM glu-1-fibrinopeptide peptide mass standard (Glu-Fib; Protea Biosciences, Inc., Morgantown, WV)] was added. Two μL of sample matrix was spotted onto a ground steel target plate (MTP 384 ground steel T F plate, Bruker Daltonics). Matrix assisted laser desorption ionization time of flight (MALDI-TOF) spectra were acquired using a Bruker AutoflexIII. Each spectra is the sum of 5,000 shots with the laser moving every 1,000 shots across a polygon pattern. A calibration mixture from the manufacturer (Bruker Daltonics) was used to calibrate the instrument during acquisition (every three to eight samples) to correct for spatial and temporal drift. Resolution was determined using the Glu-Fib internal standard and was 3200 with an average mass error of 148 ppm.

### Data processing and analysis

Statistics on haematologic and serum biochemistry values were performed using SigmaPlot (v. 11.2) to compare two groups. A *T*-test was used to determine differences between two groups, with normality first being evaluated using a Shapiro-Wilk test (α = 0.05). Non-normal data was evaluated using a Mann-Whitney Rank Sum test (α = 0.05). Raw MALDI-TOF spectra were processed using Progenesis MALDI (Nonlinear USA Inc., Durham, NC). Spectra were pre-processed using a noise filter size of 5 and background subtracted using a top hat filter size of 200. Spectra were aligned using a search area of 5 and 20 iterative cycles. Peaks with a weighted average above 1500 cps were automatically selected and manually inspected for inclusion. Peak intensities were normalized to the internal standard (Glu-Fib) or Total Ion Current (TIC) and analyzed separately based on normalization procedure. If TIC was used, Glu-Fib peaks were removed before importing into Progenesis MALDI. The independent test set was aligned with the training set to ensure proper peak alignment. These data were used for downstream analyses. Additionally, Matlab (MathWorks R2010b, Natick, MA) was used to perform unsupervised hierarchical clustering analysis of the TIC normalized peak data from the training set.

### Receiver operator characteristic (ROC) curve analysis

Receiver operator characteristic curves were generated and the area under the curve (AuROC) was determined using Matlab. A 95% confidence interval for each AuROC was determined by calculating the standard error according to Hanley et al. [34] with α = 0.05. Four different thresholds were calculated: minimum mis-classified threshold (minMC), optimum threshold (OpT), 100% negative predictive value (NPV; proportion of predicted negatives which are true negatives) threshold (npvT) and 100% positive predictive value (PPV; proportion of predicted positives which are true positives) threshold (ppvT). The minMC is the threshold that minimizes the proportion of false-negatives and false-positives. The OpT is the geometrically determined threshold which is the closest point to 100% sensitivity and 100% specificity which corresponds to the perfect test [35]. The npvT and ppvT are the thresholds with the highest specificity and 100% NPV or the highest sensitivity and 100% PPV, respectively.

### Artificial neural network analysis

The artificial neural network (ANN) algorithm was trained using Glu-Fib peptide or TIC normalized peak data from the training dataset. These training sets were used independently to train 101 feed-forward ANNs performed by Matlab as previously described [36]. Briefly, each ANN was pursued to an early stop criteria determined by internal testing using bootstrapped cross-validation with every seventh sample, in addition to screening for optimal topology. The median performing ANN, determined using AuROC values, were selected for qualification with the independent test set. In the case where the median ANN shared identical AuROC values, both were selected for qualification. It is important to note that, to avoid model over-fitting, *a priori *threshold values determined by ROC analysis of the training set were utilized as decision threshold values for qualification of the test set.

In a separate analysis, we determined statistical performance measures for each of the 101 ANNs using the test dataset for all thresholds (minMC, OpT, npvT, ppvT) and both normalization procedures (TIC and Glu-Fib), which resulted in a total of 808 tests. The ANN output was averaged across the 101 ANNs for each sea lion and was referred to as Combinatorial ANN (CANN_101_). *A priori *threshold values for qualification of the test set by CANN_101 _models were derived by ROC analysis of the averaged ANN output. In addition to CANN_101 _we developed a scheme to allow each of the 101 ANNs to "vote". This was accomplished by converting the ANN outputs > 0.5 to 1's and summing the values for each sea lion. If the sum of the 101 ANNs for a sea lion was > 50, then that sea lion was predicted to be DAT.

## Abbreviations

ANN: artificial neural network; AuROC: area under the receiver operator characteristic curve; DA: domoic acid; DAT: domoic acid toxicosis; MALDI-TOF: matrix assisted laser desorption ionization-time of flight; MMP: US Navy Marine Mammal Program; NPV: negative predictive value; PPV: positive predictive value; TMMC: The Marine Mammal Center.

## Competing interests

The authors declare that they have no competing interests.

## Authors' contributions

BN contributed to the experimental design, carried out the MALDI-TOF studies/data analysis and drafted the manuscript. JS selected serum samples used in the study, coordinated clinical data, and assisted in the draft the manuscript. DG participated in serum sample collection, coordinated/interpreted clinical data, and assisted in drafting the manuscript. KC participated in serum collection, serum selection, and coordinated clinical data for the NAVY samples. EF participated in optimization of MALDI-TOF profiling, data analysis and contributed to the methods section. FG participated in the design of the study, interpreted the diagnosis as well as clinical data and contributed to the introduction and discussion of the data. MJ conceived of the study, participated in its design and coordination, participated in the data analysis, and helped to draft the manuscript. All authors read and approved the final manuscript.

## Supplementary Material

Additional file 1 **Table S1**. Descriptive data of sea lions in the training dataset. Trauma included lacerations, bite wounds, broken bones, and anophthalmia. Pneumonia included verminous and/or parafilaroides. **Table S2**. Descriptive data of sea lions in the independent test set. Trauma was only to the flipper, and unknown was best described as lethargic. Pneumonia included parafilaroides. Empty categories under 'Primary Etiology' given to serve as a reference to training set descriptive data. **Table S3**. Haematologic and serum biochemistry data of sea lions in the independent test set. Blood chemistry data is based on analysis of blood drawn the same day as serum samples used for analysis. A *T*-test (or rank sum test if distribution was non-normal, indicated by '*') was used to compare blood chemistry values. Click here for file

Additional file 2 **Table S4**. Qualified performance of peak 3017 m/z using an independent test set. DAT is indicated by a '1' and non-DAT a '0'. Performance against training set is given for comparison. **Table S5**. Qualified performance of ANNs trained with Glu-Fib normalized data. Performance was qualified using an independent test set. DAT is indicated by a '1' and non-DAT a '0'. Performance against training set is given for comparison. **Table S6**. Qualified performance of ANNs trained with TIC normalized data. Performance was qualified using an independent test set. DAT is indicated by a '1' and non-DAT a '0'. Performance against training set is given for comparison. Click here for file

Additional file 3 **Figure S1**. Peak 1362 m/z spectra, ROC curve and performance. (A) 1362 m/z peak height was plotted between two groups. The dotted line corresponds to the threshold determined by ROC curve analysis. (B) An ROC curve was generated using 107 individuals in the training set and was used to determine the optimum threshold (OpT; indicated by arrow). (C) The OpT was used to determine statistical performance measures of peak 1362 m/z using the 107 individuals in the training set. (D) Statistical performance measures for peak 1362 m/z when all sea lions in the study (Training + Test) were combined. Click here for file

Additional file 4 **Figure S2**. Unsupervised clustering of MALDI-TOF profiles of the training set. Data were standardized by each peak (n = 104; rows) and represented such that the mean is 0 and standard deviation is ± around this mean. Columns are individuals (n = 107), labeled at the top with red squares for acute-DAT and blue squares for non-DAT. Click here for file
