# Supplementary material for: Serum profiling by MALDI-TOF mass spectrometry as a diagnostic tool for domoic acid toxicosis in California sea lions
Source: Proteome Sci. 2012 Mar 19;10:18. doi: 10.1186/1477-5956-10-18 (PMC3338078; doi:10.1186/1477-5956-10-18)
Supplement: Additional file 3 — Figure S1. Peak 1362 m/z spectra, ROC curve and performance. (A) 1362 m/z peak height was plotted between two groups. The dotted line corresponds to the threshold determined by ROC curve analysis. (B) An ROC curve was generated using 107 individuals in the training set and was used to determine the optimum threshold (OpT; indicated by arrow). (C) The OpT was used to determine statistical performance measures of peak 1362 m/z using the 107 individuals in the training set. (D) Statistical performance measures for peak 1362 m/z when all sea lions in the study (Training + Test) were combined. [file 1477-5956-10-18-S3.PDF]

A B

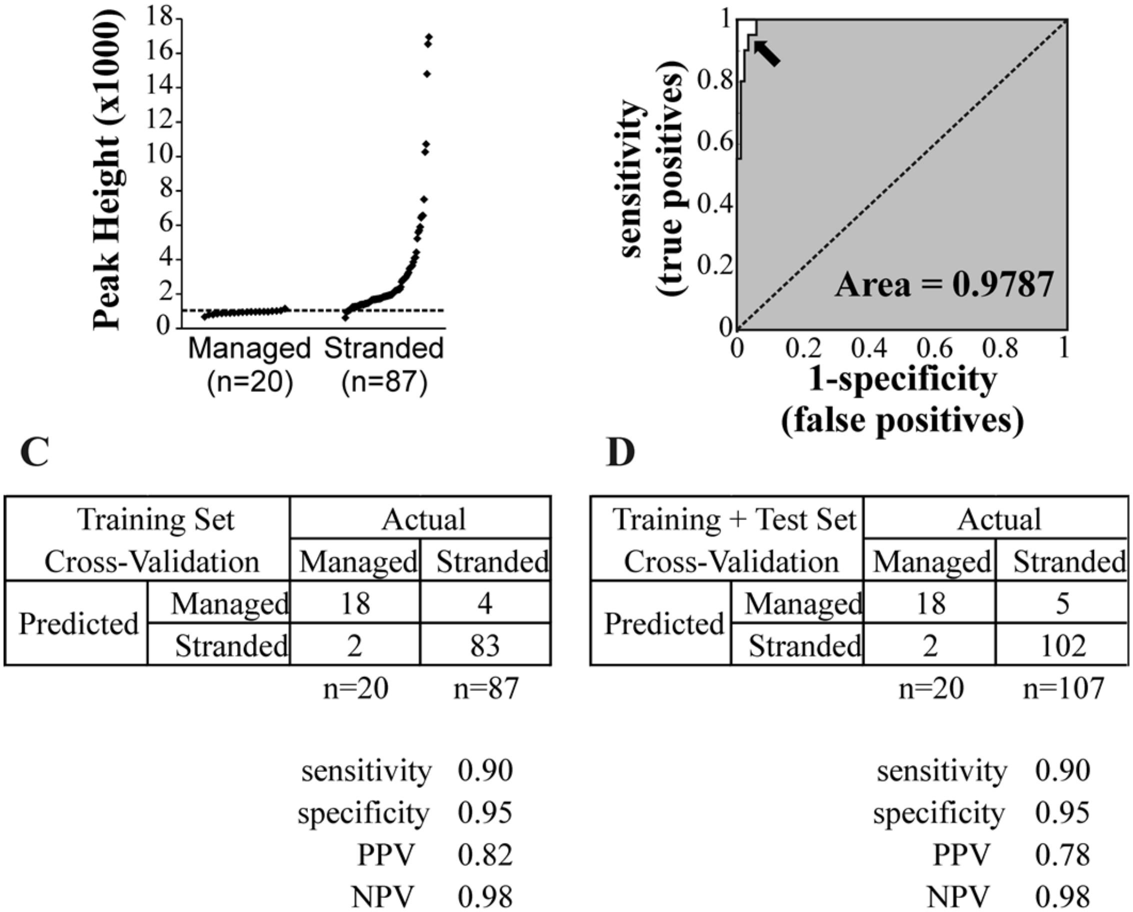

C

Training Set Cross-Validation		Actual	
		Managed	Stranded
Predicted	Managed	18	4
	Stranded	2	83
		n=20	n=87

sensitivity	0.90
specificity	0.95
PPV	0.82
NPV	0.98

D

Training + Test Set Cross-Validation		Actual	
		Managed	Stranded
Predicted	Managed	18	5
	Stranded	2	102
		n=20	n=107

sensitivity	0.90
specificity	0.95
PPV	0.78
NPV	0.98

Figure S1. Peak 1362 m/z spectra, ROC curve and performance. (A) 1362 m/z peak height was plotted between two groups. The dotted line corresponds to the threshold determined by ROC curve analysis. **(B)** An ROC curve was generated using 107 individuals in the training set and was used to determine the optimum threshold (OpT; indicated by arrow). **(C)** The OpT was used to determine statistical performance measures of peak 1362 m/z using the 107 individuals in the training set. (**D**) Statistical performance measures for peak 1362 m/z when all sea lions in the study (Training + Test) were combined.
