# Supplementary material for: Serum profiling by MALDI-TOF mass spectrometry as a diagnostic tool for domoic acid toxicosis in California sea lions
Source: Proteome Sci. 2012 Mar 19;10:18. doi: 10.1186/1477-5956-10-18 (PMC3338078; doi:10.1186/1477-5956-10-18)
Supplement: Additional file 4 — Figure S2. Unsupervised clustering of MALDI-TOF profiles of the training set. Data were standardized by each peak (n = 104; rows) and represented such that the mean is 0 and standard deviation is ± around this mean. Columns are individuals (n = 107), labeled at the top with red squares for acute-DAT and blue squares for non-DAT. [file 1477-5956-10-18-S4.PDF]

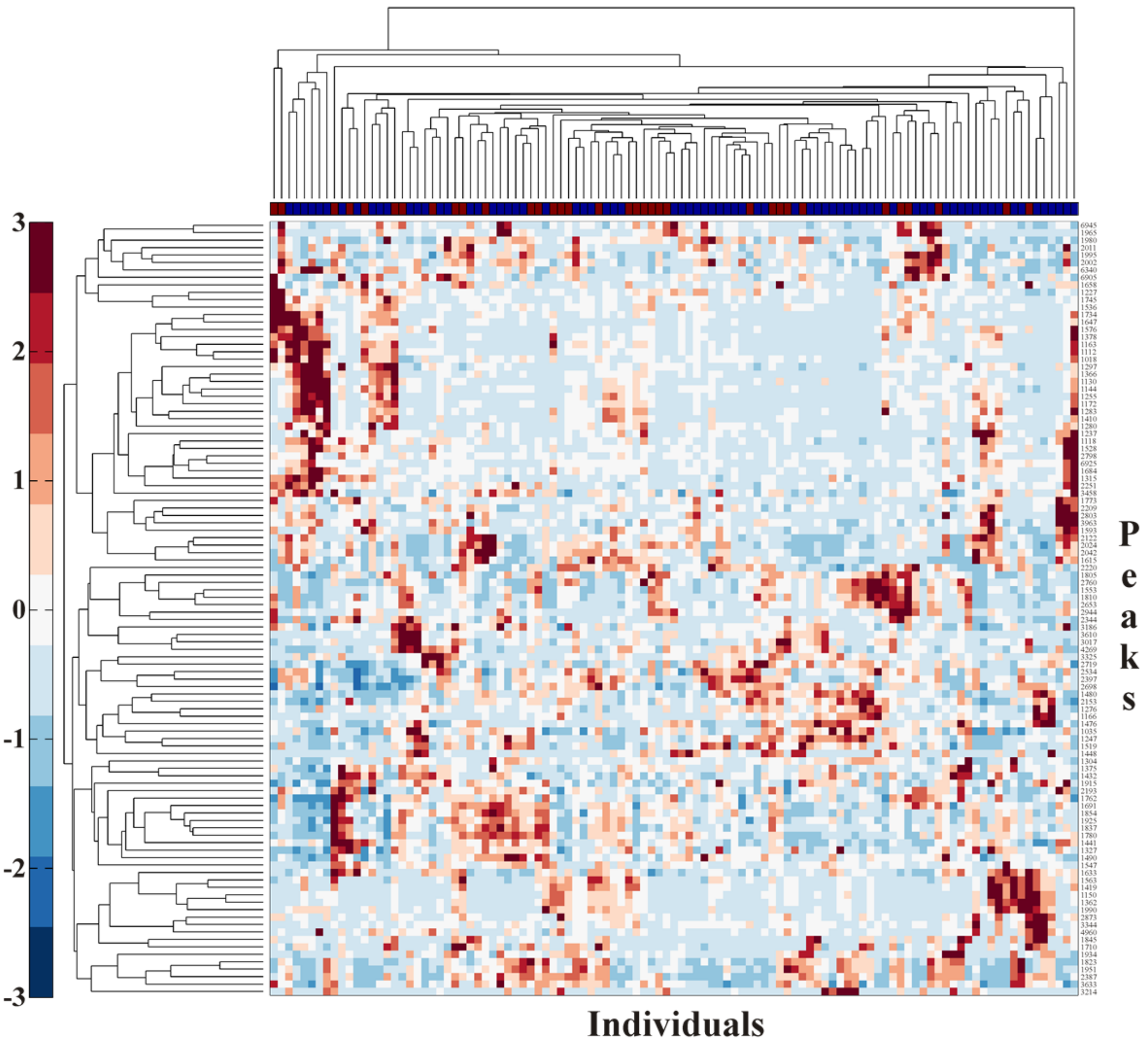

Figure S2. Unsupervised clustering of MALDI-TOF profiles of the training set. Data were standardized by each peak (n=104; rows) and represented such that the mean is 0 and standard deviation is ± around this mean. Columns are individuals (n=107), labeled at the top with red squares for acute-DAT and blue squares for non-DAT.
